# Developing a Digital Health Intervention for Conversation Skills After Brain Injury (convers-ABI-lity) Using a Collaborative Approach: Mixed Methods Study

**DOI:** 10.2196/45240

**Published:** 2023-08-09

**Authors:** Petra Avramović, Rachael Rietdijk, Belinda Kenny, Emma Power, Leanne Togher

**Affiliations:** 1 Discipline of Speech Pathology Faculty of Medicine and Health The University of Sydney Camperdown Australia; 2 Discipline of Speech Pathology Faculty of Medicine and Health The University of Sydney Sydney Australia; 3 Discipline of Speech Pathology School of Health Sciences Western Sydney University Sydney Australia; 4 Discipline of Speech Pathology Graduate School of Health University of Technology Sydney Sydney Australia

**Keywords:** brain injury, cognitive-communication, communication partner training, digital health, co-design

## Abstract

**Background:**

People with acquired brain injury (ABI) experience communication breakdown in everyday interactions many years after injury, negatively impacting social and vocational relationships. Communication partner training (CPT) is a recommended intervention approach in communication rehabilitation after ABI. Access to long-term services is essential, both in rural and remote locations. Digital health has potential to overcome the challenges of travel and improve cost efficiencies, processes, and clinical outcomes.

**Objective:**

We aimed to collaboratively develop a novel, multimodal web-based CPT intervention (convers-ABI-lity) with key stakeholders and evaluate its feasibility for improving conversation skills after brain injury.

**Methods:**

This mixed methods study consisted of 3 key stages guided by the Integrate, Design, Assess, and Share (IDEAS) framework for developing effective digital health interventions. Stage 1 included the integration of current end-user needs and perspectives with key treatment and theoretical components of existing evidence-based interventions, TBI Express and TBIconneCT. Stage 2 included the iterative design of convers-ABI-lity with feedback from end-user interviews (n=22) analyzed using content analysis. Participants were individuals with ABI, family members, health professionals, and paid support workers. Stage 3 included the evaluation of the feasibility through a proof-of-concept study (n=3). A total of 3 dyads (a person with ABI and their communication partner [CP]) completed 7 weeks of convers-ABI-lity, guided by a clinician. The outcome measures included blinded ratings of conversation samples and self-report measures. We analyzed postintervention participant interviews using content analysis to inform further intervention refinement and development.

**Results:**

Collaborative and iterative design and development during stages 1 and 2 resulted in the development of convers-ABI-lity. Results in stage 3 indicated positive changes in the blinded ratings of conversation samples for the participants with traumatic brain injury and their CPs. Statistically reliable positive changes were also observed in the self-report measures of social communication skills and quality of life. Intervention participants endorsed aspects of convers-ABI-lity, such as its complementary nature, self-guided web-based modules, clinician sessions, engaging content, and novel features. They reported the intervention to be relevant to their personal experience with cognitive-communication disorders.

**Conclusions:**

This study presents the outcome of using the IDEAS framework to guide the development of a web-based multimodal CPT intervention with input from key stakeholders. The results indicate promising outcomes for improving the conversation skills of people with ABI and their CPs. Further evaluation of intervention effectiveness and efficacy using a larger sample size is required.

## Introduction

### Background

Cognitive-communication disorders following acquired brain injury (ABI) [1,2] can substantially disrupt a person’s ability to communicate and form and maintain relationships with others [3] across their life trajectory [4]. Disruptions in high-level cognitive-linguistic function [1] lead to conversation difficulties in the domains of topic generation, conversation initiation, inappropriate comments, verbosity, reading and using nonverbal cues, and the complexity of managing simultaneous cognitive and communication demands [5]. Such difficulties can have a considerable impact following injury on family functioning and psychological well-being for several years following the injury [6]. Communication changes were reported to be one of the primary issues that place strain on intimate relationships after ABI [7], and family members reported changes in social cognition, insight, and self-monitoring as key areas of distress [6]. People with cognitive-communication disorders have overall poorer outcomes in the domains of relationships, work, and leisure [8] and reduced quality of life due to declining social circles [9] and job stability [10] than people without cognitive-communication disorders.

Communication partner training (CPT) approaches are often recommended as an intervention in communication rehabilitation after ABI [11-14]. CPT is an essential component during recovery and rehabilitation to maintain relationships after ABI and contributes to improving outcomes for people with ABI and their families [15] by actively involving friends and family members in the rehabilitation process [16]. There are several evidence-based CPT programs available for people with chronic ABI and their communication partners (CPs). TBI Express [17,18] is a well-recognized program for people with severe ABI and their close CPs, with evidenced improvements in everyday communication abilities [18]. CPT has also been implemented to train paid support workers who work with people with ABI. Training support workers had a positive effect on improving conversational interactions for people with ABI, which led to increased independence for the person with ABI both in their long-term care facility and their community [19]. Following CPT, support workers also reported increased knowledge and use of strategies, improved communication with their clients, and more positive emotional experiences [20]. Existing interventions such as TBI Express [17,18] have limitations that may impede clinical use, including being a high-dose treatment (35 hours over a 10-week period) delivered via face-to-face individual and group formats using a static paper-based manual [21]. Strategies to enhance clinical implementation may include reducing the number of direct clinician hours, increasing the flexibility of service delivery, and developing web-based options.

Advancements in technology have the potential to offer new formats of delivering information and, therefore, new opportunities for greater treatment success in ABI. There are benefits of technology in promoting and maintaining social connections, vocation, and education, which have been accentuated throughout the COVID-19 pandemic [22]. The growth of digital health modalities has facilitated the development of intervention programs that give people with ABI timely and cost-effective access to specialized services, allowing them to increasingly take control of and play a more active role in their health [23]. An example of such a digital intervention is TBIconneCT [24,25], a CPT program developed after feedback about the face-to-face program, TBI Express [17,18]. TBIconneCT was developed in response to barriers that affected delivery of the group-based components of TBI Express. In addition to geographic constraints, most family members of patients with ABI have competing time demands, making weekly attendance at a rehabilitation center difficult. To overcome these challenges, the group component was removed, direct clinician hours were reduced, and web-based videoconferencing sessions were introduced [25] in TBIconneCT. Rietdijk et al [26] found that there were no substantial differences between telehealth and in-person participants in retention rate, program completion time, degree of home practice completion, or therapeutic alliance ratings. Primary outcome measures also demonstrated improvements in conversation skill support and participation in TBIconneCT compared with TBI Express. Participants described telehealth delivery as opening “a window for access to rehabilitation in the context of my daily life” [26]. TBIconneCT offers a telehealth alternative for the delivery of CPT to people with ABI and their families. People with ABI have the opportunity to apply, internalize, and practice skills and tasks within their own homes during therapy sessions and receive real-time feedback from their clinician [27]. As technology continues to advance and new digital features become available for integration into digital interventions, there is potential to evolve existing evidence-based interventions to increase flexibility in service delivery and provide clinicians, people with ABI, and families with additional asynchronous, treatment options. With interventions transitioning into the digital space, it is essential to maintain the theoretical underpinnings that preceding evidence-based interventions were built upon [28].

In a recent systematic review of the effectiveness of ABI rehabilitation, digital health interventions, and their key characteristics, only 9 out of 44 studies stated the underpinning theoretical models of the interventions [28]. Regarding CPT interventions, TBI Express and TBIconneCT have been based on the theoretical foundations of cognitive, behavioral, and educational theory [29]. They facilitate conversation skill development through strategies, such as role-playing and modeling, in adjunct with coaching and support from the clinician [30,31]. Meulenbroek et al [32] identified that behavioral and cognitive treatment theories are integral in social communication treatments to ensure effectiveness of the treatment. Ensuring that innovative digital adaptations of existing CPT programs maintain the foundations of behavioral and cognitive treatment theories will facilitate the transference of positive communication outcomes into new intervention iterations and support conversational skill generalization across the International Classification of Functioning [33] domains of impairment, activity, and participation. Alongside using existing knowledge as a foundation for intervention design and development, a collaborative approach is recommended to ensure that digital health interventions fulfill end-user needs and wants [34]. In the aforementioned systematic review of current digital health interventions for people with ABI and their caregivers, only 5 interventions involved elements of co-design [28]. There has been limited exploration of end-user perspectives during the development process of CPT interventions and other digital health applications addressing cognitive and behavioral impairments after ABI [28]. CPT interventions in ABI rehabilitation involve a variety of end users, including people with ABI, their family members and friends, speech pathologists, paid support workers, and other health professionals. A recent study (Avramovic, P, unpublished data, December 2022) exemplifies that each group of end users present with individual and nuanced perspectives on the management of cognitive-communication disorders, which can be incorporated into the design of intervention content and features. From this study (Avramovic, P, unpublished data, 2022), four themes arose from participant interviews: (1) brain injury changes the way we communicate; (2) conversations after a brain injury can be challenging for both of us; (3) in positive conversations, we aim to make it equal; and (4) the nature of brain injury requires tailored education and knowledge and skills training. Participants highlighted that the heterogeneity of brain injury and cognitive-communication disorders calls for an individualized and engaging approach to communication education and training. Through the integration of the perspectives, wants, and needs of people with ABI in the co-design of cognitive-communication–based interventions, end users may benefit by receiving more meaningful services with the possibility of reduction in development costs and time.

Co-design has recently become a critical focus for intervention development across the health sector to improve outcomes and recognize the expertise and value of involving end users in service and intervention development [35]. There are various discourses and terminologies [36], but a central definition is that co-design is an approach whereby the end users form part of the collaborative design team as “experts of their experiences” [37] throughout the duration of the design process [37-39]. The agreed underlying co-design values and principles are notions of equal and reciprocal relationships between professionals and end users [40] that are achieved through respect, support, transparency, responsiveness, fairness of opportunity, and accountability from all those involved [41]. There are differing levels of involvement in co-design that range from roles of listeners to decision makers [42], which may be applied to the entire research process or at specific stages. Through their systematic review, Masterson et al [36] urge that future research should focus on applying the underlying principles and values of co-design, rather than seeking universal definitions for the various terms associated with co-design. The Integrate, Design, Assess, and Share (IDEAS) [43] framework has been developed to guide the development of effective digital interventions with end-user involvement. This framework consists of the integration of behavioral theory, design thinking, user-centered design, rigorous evaluation, and dissemination of findings. A detailed and collaborative approach across the course of development can maximize the relevance, content, and delivery methods for uptake of the program.

### Objectives

This paper had 2 aims. The first aim was to describe the collaborative development process of adapting the TBI Express and TBIconneCT programs to a new digital platform called convers-ABI-lity, which was guided by the perspectives and experiences of end users. The second aim was to evaluate the use of the digital platform to deliver the convers-ABI-lity intervention in a proof-of-concept study of 3 participant dyads to address the following research questions:

1. Does convers-ABI-lity produce changes in conversations between the person with ABI and their CP as measured by objective conversation rating scales?
2. Does convers-ABI-lity produce changes on self-report and significant other–report measures of social communication?
3. Does convers-ABI-lity produce changes to quality of life and participation in everyday life as measured by a self-report quality of life measure?
4. Was it feasible to deliver convers-ABI-lity using a digital health platform?
5. How was convers-ABI-lity perceived by participants?

## Methods

### Overview

A sequential, exploratory mixed methods design [44], guided by the phases of the IDEAS framework [43], was used to develop convers-ABI-lity, a digital health intervention for improving conversation skills after ABI. The Good Reporting of a Mixed Methods Study checklist [45] was used to guide the reporting of this study (Multimedia Appendix 1 [45]). This paper focuses on phases 1 to 8 of the framework, which have been divided into 3 stages (Table 1). Stage 1 sought to establish intervention content, principles, and theories with the integration of user insights and their lived experience. Stage 2 involved rapid and iterative design of the intervention with user feedback. Stage 3 investigated the feasibility of the intervention in a proof-of-concept study with 3 participants with brain injury and their CPs.

The digital health intervention called convers-ABI-lity is a component of a larger body of work being developed by the ABI Communication Lab at the University of Sydney, called the Social Brain Toolkit. The Social Brain Toolkit aims to support people with ABI, their family members and friends, support workers, and health professionals involved in the care of a person with ABI to have more positive interactions and conversations in person and via web-based platforms. It consists of three digital education, training, and intervention components: (1) interact-ABI-lity, (2) social-ABI-lity, and (3) convers-ABI-lity. The Social Brain Toolkit project team consisted of speech pathologists and other allied health clinicians, researchers, and rehabilitation funders. The project was also guided by the Social Brain Toolkit Advisory Committee, consisting of a person with an ABI, his family member, a speech pathologist working in a regional brain injury rehabilitation service, a speech pathologist working in private practice, a representative from the NSW Brain Injury Rehabilitation Program, and a representative from eHealth NSW. Proceeding in parallel to this study, the advisory committee provided input on 6 occasions during the development of the project and provided input on the research and development of the Social Brain Toolkit, including convers-ABI-lity.

A collaborative approach was undertaken during the planning, design, and development stages of convers-ABI-lity. Principles of co-design [36] such as building on individuals’ existing capabilities, reciprocity, and mutuality and engaging others to transfer knowledge were applied throughout development. The level of involvement of the stakeholders involved is described through the Involvement Matrix [42] presented in Table 2.

**Table 1:** An overview of the project stages, Integrate, Design, Assess, and Share (IDEAS) framework phases, and objectives.

Stage	IDEAS [43] framework phases	IDEAS [43] phase description	Objectives
1	Empathize with target users Ground in behavioral theory Specify target behavior	Collect stakeholder perspectives and lived experience Underpin intervention with theory and existing evidence Define specific behaviors that the intervention will target	Adapt core content of existing evidence-based interventions while maintaining theoretical models in a digital environment Integrate findings from previously conducted qualitative interviews with adapted core content to inform ideation of intervention modules and tasks
2	Ideate implementation strategies Prototype potential products Gather user feedback Build an MVP^a^	Brainstorm novel strategies for integrating theory, evidence, and end-user perspectives into content and features Rapidly and iteratively build prototypes of intervention Seek end-user feedback and insights on prototypes Develop the MVP with enough functionality for trial of intervention	Generation and early testing of intervention modules, tasks, and features Collect and analyze feedback from end users on features and content Develop an MVP for feasibility testing
3	Evaluate early intervention outcomes and usability	Conduct small-scale evaluation to determine, efficacy, feasibility, and end-user experiences and perspectives	Evaluate the feasibility of the intervention platform Gather user feedback on usability and satisfaction Further modify the platform based on user feedback

^a^MVP: minimum viable product.

**Table 2:** Levels of involvement.

Stakeholder category	Level of involvement	Definition of level of involvement
Project team	Decision makers	Lead the decision-making across the project
Advisory committee	Partner or adviser	Works as an equal partner with joint decision-making and is asked for opinions regularly and informed how the opinion has been used
Research participants	Cothinkers	Invited to provide opinions or complete a task (opinions may or may not be adopted)

### Recruitment

Participants were recruited via posts on social media (Twitter and Facebook), speech pathology and brain injury rehabilitation email networks of the researchers, and community case managers at a metropolitan brain injury rehabilitation unit. Participants completed an Assessment of Capacity to Consent [46] form developed specifically for this study during videoconference (Zoom) meetings with authors PA or RR. Participants were provided with the opportunity to discuss the study and ask questions. Written consent was provided by all participants before participating in the research. Participants included in stage 2 of this study had previously participated in a study (Avramovic, P, unpublished data, 2022) investigating the perspectives, needs, and wants of people with ABI and their supporters in regard to their experiences of cognitive-communication disorders, with the aim of developing a digital health intervention. Participants in stage 3 (proof-of-concept evaluation) of this study had not participated in the previous phases of the project.

### Inclusion and Exclusion Criteria

Participants were required to be ≥18 years of age. The inclusion and exclusion criteria for each participant group are provided in Textbox 1.

Family members or usual CPs were eligible to participate if they interacted with a person with ABI at least once a week, had known the person with ABI for at least 3 months, or had not sustained a severe ABI. Speech pathologists and other health professionals (including paid support staff) were eligible to participate if they were employed in a clinical role working with people with ABI for at least 2 years or equivalent. Participants in stage 3 (proof-of-concept evaluation) of the study were recruited via social media (Twitter and Facebook) posts, speech pathology networks, and brain injury rehabilitation networks. The inclusion and exclusion criteria were the same as for participants in stage 2 (design iteratively and rapidly with user feedback). Only participants with ABI and a usual CP were involved in the proof-of-concept study.

**Inclusion and exclusion criteria.**

**Inclusion criteria for participants with acute brain injury**

- A moderate-severe traumatic brain injury at least 6 months before the study. This was based on the Mayo classification scheme [47] where at least one of the following was applicable: loss of consciousness of >30 minutes, posttraumatic amnesia of >24 hours, worst Glasgow Coma Scale total score in the first 24 hours of <13, or evidence of a major brain imaging abnormality. People with nontraumatic brain injuries (stroke, hypoxic brain injury, brain tumor, poisoning, or infection) were also eligible for this study.
- Spent time at home on a regular basis and was discharged fully or partially from the hospital.
- Substantial social communication skills deficits.
- Insight into their social communication skills deficits.
- Adequate English proficiency without the aid of an interpreter for completing assessment tasks.
- Reading skills that were functional in English.

**Participants were excluded if they had a presence of the following:**

- Aphasia that prevented conversational participation.
- Severe amnesia that prevented provision of informed consent.
- Substantially reduced intelligibility in conversation due to dysarthria.
- Drug or alcohol addiction preventing reliable participation in sessions.
- Active psychosis.
- Premorbid intellectual disability, ≥1 episode of moderate to severe brain injury or cooccurring degenerative neurological disorder.

### Procedure

**Figure 1:**
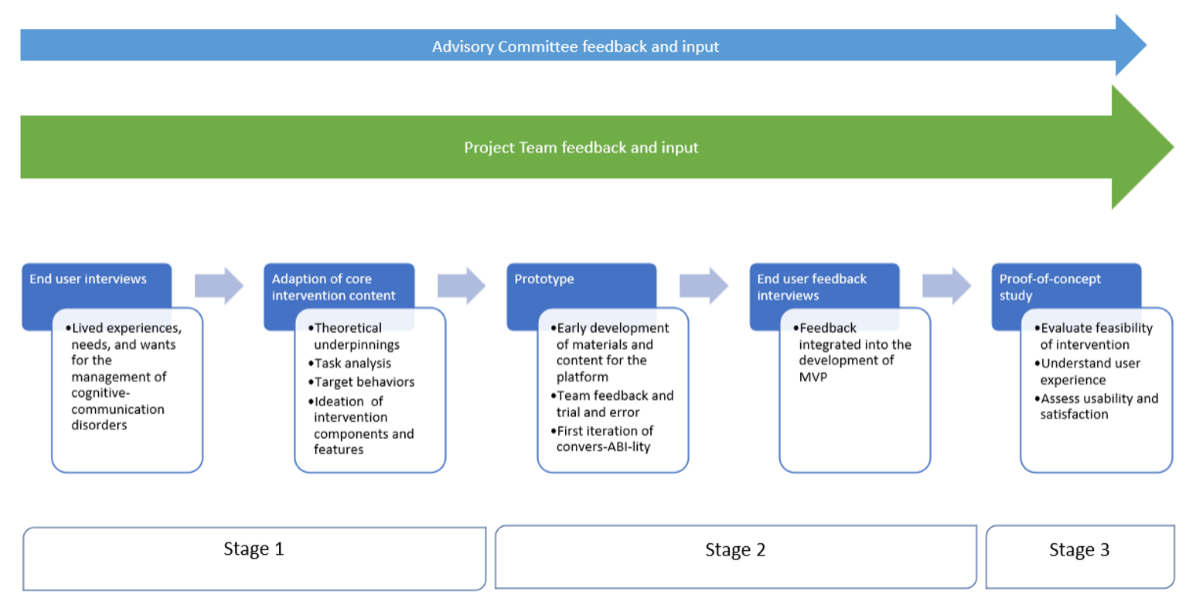
Overview of development stages. MVP: minimum viable product.

Figure 1 depicts the tasks undertaken in each phase of this research.

#### Stage 1: Integrate Insights From Users and Theory

In stage 1, the project team participated in monthly reflective and brainstorming workshops to identify the core components of existing evidence-based programs, TBI Express [48] and TBIconneCT [49]. The team identified content that can be delivered asynchronously and synchronously; adapted intervention components into a web-based modality; and refined early prototypes of intervention content, materials, and tasks. Throughout the adaptation process, the research team ensured that the underlying theoretical models of behaviorism [50], cognitive theory [51], and education theory were retained as the content was transferred into a web-based and digital environment. This process was further informed by qualitative data gathered from participants of previous TBI Express and TBIconneCT studies and thematic analysis of participant perspectives and experiences with cognitive-communication disorders from previously conducted semistructured interviews [21]. Participants in a previously reported study (Avramovic, P, unpublished data, 2022) also shared lived experiences of cognitive-communication disorders and recommendations for the development of digital resources for improving conversations. Three members of the research team were also involved in the development of TBI Express and TBIconneCT (LT, EP, and RR).

#### Stage 2: Design Iteratively and Rapidly With User Feedback

In stage 2, the research team built the first iteration of the prototype platform for delivery of convers-ABI-lity. The modules, activities, and resources were reviewed and tested by the research team members, resulting in the final prototype used to gather feedback from end users. The participants were interviewed via videoconference (Zoom) by authors PA and RR, from November 2020 to February 2021. PA and RR are speech pathologists with experience working with people with ABI and their family members. The participants were known to the researchers from the previous semistructured interviews (Avramovic, P, unpublished data, 2022), where they shared their lived experiences of cognitive-communication disorders. Interviewing the same cohort of participants supported the iterative nature of this collaborative project. Each participant completed a 1-hour interview to provide feedback on the features and content of the Social Brain Toolkit. However, this paper will report only the content of the interview relevant to the development of convers-ABI-lity. Interviewers used a cognitive interviewing [52] approach, drawing upon aspects of the “think-aloud” method to facilitate the interviews by providing participants with general instruction or questions, followed by more specific prompts [53]. The topics covered in the interview are provided in Multimedia Appendix 2. The interview was supported with the use of visuals to aid comprehension and attention depicted via presentation slides that were screenshared on Zoom. Following the interview, the participants were emailed a summary of key points discussed during the interview and were provided with opportunities to member-check their responses.

#### Stage 3: Proof-of-Concept Evaluation

Stage 3 focused on investigating the feasibility and usability of completing convers-ABI-lity using the prototype platform. This was conducted via a proof-of-concept study, which included 3 dyads as participants consisting of a person with severe ABI and a usual CP. Participants completed convers-ABI-lity, a conversation skills intervention program consisting of seven 1-hour videoconference sessions from home, guided by an experienced and qualified speech pathologist (author RR). The sessions were scheduled weekly and self-guided web-based modules were completed between sessions. All 3 dyads were loaned an iPad (Apple Inc) with prepaid internet data access to use for the duration of the intervention. On the completion of the intervention, participants took part in a semistructured qualitative interview via videoconferencing on the nature of the project and their experience (Multimedia Appendix 3) with an independent interviewer.

### Data Collection and Analysis

#### Stage 1: Integrate Insights From Users and Theory

Research team workshops were video recorded. PA collected field notes during meetings detailing the team decision-making processes and RR maintained a record of the team decisions during the adaptation of core content and the ideation phase of new content and material. Early examples of content such as tasks, activities, videos, and audio were circulated to all team members for feedback, which were collected and synthesized by RR to inform the development of the initial prototype.

#### Stage 2: Design Iteratively and Rapidly With User Feedback

##### Injury Severity Characteristics

The level of disability and recovery for each participant with traumatic brain injury (TBI) was rated using the Glasgow Outcome Scale–Extended (GOS-E) [54]. The Care and Needs Scale (CANS) [55] was administered to understand the level of self-care support an individual with ABI requires. To assess participant cognitive-communication skills, a portion of the Functional Assessment of Verbal Reasoning and Executive Strategies (FAVRES) [56] was administered. Only Task 4 of the FAVRES was administered as this task has the strongest correlations to the cognitive domains of attention, processing speed, memory, and executive function [57]. Furthermore, this task was the most feasible to administer via videoconference as the participant booklet was the only required test material. Limited permission for the use of the FAVRES via videoconference was provided by CCD publishing, ensuring that steps were taken for consistent test administration, test integrity, and respect of copyright.

##### Participant Interviews

Participant interviews were video recorded with participant consent (documented on each participant consent form), using the videoconferencing feature. Verbatim transcriptions were then completed for all interviews. Due to the step-by-step and demonstrative nature of the interview that allowed for checking and clarification, the transcripts were not provided to the participants. Interviewers also collected field notes during and after the interview.

Transcriptions were analyzed using conventional content analysis [58]. Data were analyzed by PA who is a female speech pathologist with experience working with people with ABI and their family members. PA is currently a full-time PhD candidate in the field of communication after brain injury, with previous experience in qualitative interview data analysis. The analysis process consisted of 4 phases. In phase 1, PA familiarized herself with the data set by reading and rereading the transcriptions, with reference to the original recording. Phase 2 consisted of parsing the transcriptions into meaning units in Microsoft Excel and applying an inductive approach to data coding to ensure that the codes were generated from the data. This process is detailed in Multimedia Appendix 4. Initial codes were then refined by revisiting the transcriptions and through peer checking to ensure the accuracy of code titles. Consensus through discussion with the research team was reached for all codes. In phase 3, codes were grouped into subcategories based on semantic similarities and a label was assigned based on consensus. In phase 4, subcategories were collated into categories. Methodological rigor was ensured through close familiarization with the data set, regular peer checking, and discussions throughout the analysis, and audit trails were maintained, documenting rationales for decision-making throughout the research and analysis process, including the documentation of debriefing with research team members. Participants were not involved in reviewing the transcripts or data analysis. Participant demographic variables and assessment scores were analyzed using descriptive summaries.

#### Stage 3: Proof-of-concept Evaluation

Participants in phase 3 completed the descriptive injury characteristic measures as outlined in phase 2. These measures included the GOS-E, CANS, and Task 4 of the FAVRES. In addition to these measures, participants in phase 3 completed the Repeatable Battery for the Assessment of Neuropsychological Status (RBANS) [59]. This assessment consists of 12 subtests measuring attention, language, visuospatial or construction abilities, and immediate and delayed memory and is reliable for administration via videoconference [60].

### Outcome Measures

Outcome measures in the proof-of-concept study included preintervention, postintervention, and follow-up blinded ratings of casual and purposeful conversation samples using the Adapted Kagan Scales [61] and self-report measures. For the casual conversation, participants were asked to have a conversation about any topic for 8 minutes, and for the purposeful conversations, participants were asked to have a conversation about an important communication event coming up in the next 4 weeks for 5 minutes. The self-report measures consisted of the La Trobe Communication Questionnaire (LCQ) [62], Sydney Psychosocial Reintegration Scale 2 (SPRS-2) [63], Quality of Life after Brain Injury (QOLIBRI) [64], and the Traumatic Brain Injury Caregiver Quality of Life (TBI-CareQOL) [65]. The casual and purposeful conversation samples were collected preintervention and postintervention, and the conversation upload tool was integrated into the self-guided modules in the prototype platform. The self-report measures were also collected via questionnaire tools embedded into the self-guided modules. The Adapted Kagan Scales [61] included the Measure of Participation in Conversation (MPC; adapted) for people with brain injury and Measure of Support in Conversation (MSC; adapted) for CPs. The scales are scored on a 9-point rating scale (0 to 4 with 0.5 increments). Further description of the scales is provided in Multimedia Appendix 5. The Adapted Kagan Scales are a recommended measure for evaluating conversation outcomes pre- and postintervention for ABI [66,67]. The scales are ecologically grounded and feasible for administration [68], as well as responsive to CP interventions [17,19,24]. Two independent assessors completed blinded ratings of video recorded casual and purposeful conversation tasks. The raters were blinded to the time points of when conversational samples were collected: preintervention, postintervention, or at follow-up. The 2 raters (PA and a research assistant) completed formal training on observation scales that took 10 hours, commencing ratings on the study samples. The training was facilitated by author RR, who is experienced in using the Adapted Kagan Scales. PA and the research assistant had prior experience using the Adapted Kagan Scales as part of their professional training and previous research projects and rated conversation samples by consensus. The ratings from the 2 independent assessors were compared among the preintervention, postintervention, and follow-up conversation samples. With possible scores ranging from 0 to 4, an improvement of 0.5 was considered a clinically meaningful change, consistent with previous studies [24,25]. The LCQ was used as a secondary outcome measure to evaluate perceived communication change. The LCQ measures the perceptions of communication skills of the person with TBI, completed by the person with ABI (self-report) and a CP (significant other–report) [62]. Both versions consist of 30 items that focus on social communication problems that occur after ABI. The person with ABI is rated on a 4-point frequency scale, with total scores ranging from 30 to 120. Higher scores indicate greater perceived difficulty. The LCQ has strong psychometric properties [62]. The SPRS-2 was used to investigate the level of participation of the person with ABI across different life domains in response to possible communication changes. The SPRS-2 evaluates the level of participation across 3 areas: work and leisure, interpersonal relationships, and living skills. The items within these 3 domains are scored on a 5-point scale, where higher scores indicate higher levels of participation. Psychometric properties are strong for interrater reliability (intraclass correlation coefficient [ICC] 0.94), test-retest reliability (ICC 0.91), and internal consistency (Cronbach =.89) [63].

The final secondary outcome measures were the QOLIBRI [64] and TBI-CareQOL [65] for measuring the quality of life and level of participation of the person with ABI and their CP. QOLIBRI comprises 37 items across 6 domains of health-related quality of life for the person with ABI. These are cognition, self, daily life and autonomy, social relationships, emotions, and physical problems. A higher score in each domain represents greater satisfaction and less problems. The scales have good test-retest reliability (ICC 0.78-0.85) and are internally consistent for each domain (Cronbach =.75-.89) [69] and have shown change in a previous study [24]. Caregiver quality of life was measured using TBI-CareQOL, which evaluated caregiver-specific quality of life across 5 key domains: anxiety, strain, loss of person with TBI, loss of self, and feeling trapped. These domains were deemed to be the most applicable ones to caregivers of people with cognitive-communication disorders following ABI. Higher scores indicate worse health-related quality of life. The internal consistency for this measure is excellent (Cronbach =.92-.93) as well as the test-retest reliability (ICC 0.92-0.94) [70].

Given the small sample size of 3 dyads, the reliable change index (RCI) [71] was used to evaluate changes from the preintervention to postintervention and from postintervention to 3-month follow-up for all self-report measures. An RCI 1.96 indicates a statistically reliable change over time for an individual score [71]. The SPRS-2 was not analyzed due to missing responses from 2 out of 3 participants, which prevented the calculation of a total score required for RCI analysis using logit scores [63]. A sample size of 3 dyads was deemed sufficient to yield meaningful results for evaluating feasibility at the proof-of-concept stage and is consistent with sample sizes reported in previous literature [25,72,73].

Process measures were collected to assist in determining the feasibility of delivering convers-ABI-lity. These measures included the number of participants retained in the program, number of sessions completed, number of weeks required to complete the intervention, and level of completion of self-guided modules. These data were analyzed descriptively. Participant interviews following intervention were video recorded with participant consent (documented on the consent form), using the videoconferencing feature. The same process as described in stage 2 (design iteratively and rapidly with user feedback) was followed for transcription and conventional content analysis.

### Ethics Approval

We obtained ethics approval from the Western Sydney Local Health District Human Research Ethics Committee (Ref: 6294—HREA 2019/ETH13510) to conduct this body of work.

## Results

### Stage 1: Integrate Insights From Users and Theory

The overall aim of stage 1 was to adapt the core content from TBI Express [48] and TBIconneCT [49] based on previous participant and key stakeholder feedback and recommendations into a digital environment while maintaining theoretical underpinnings of behavioral, cognitive, and educational theories on which TBI Express and TBIconneCT are based. The core messages and associated submessages derived from the reflective workshops are presented in Table 3. These core messages form the focus of the content and communication skills covered across the convers-ABI-lity intervention. With these core messages, the web-based intervention program (accessed via web-browser) was planned to consist of 7 asynchronous, self-guided web-based modules and 7 videoconferencing sessions with a speech pathologist. As part of their weekly self-directed tasks, it was ideated that clients would complete and upload practice conversations onto the web-based platform.

The differences and similarities in key therapeutic content and processes across TBI Express, TBIconneCT, and convers-ABI-lity for the module titled “Work Together to Get the Message Across” are listed in Table 4. One example of a key difference in content relating to communication as a cooperative process between speaker and listener is that this content is covered both synchronously and asynchronously in convers-ABI-lity compared with TBIconneCT where it is only addressed in the synchronous session. Participants watch and interact with videos describing and demonstrating collaborative approaches to conversation in the self-directed module and then consolidate this new knowledge in the videoconference session. This therapeutic process also allows for additional time to practice conversation skills and strategies during the videoconference session with clinician support and feedback as less time is spent on explaining new content. As demonstrated in Table 4, in the asynchronous session covering turn-taking, participants rate and observe how much each person talks during a recorded practice conversation as an additional learning opportunity for understanding and applying these new strategies.

**Figure 2:**
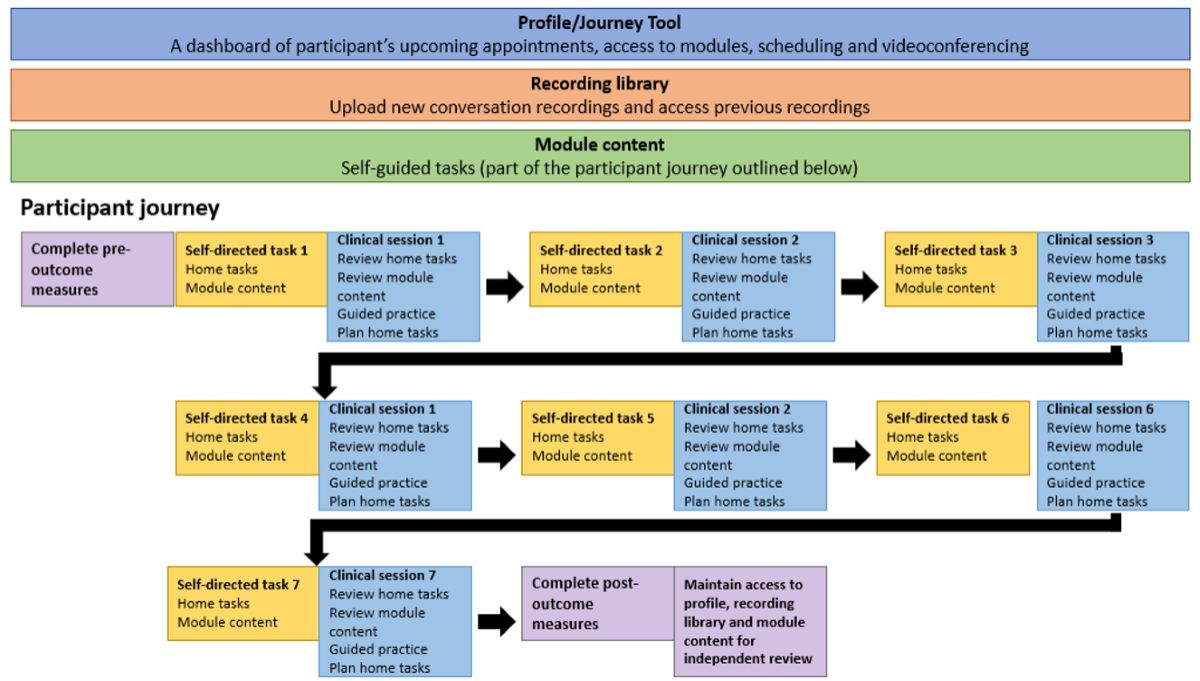
Core components of the platform.

The overall design of the convers-ABI-lity platform and participant journey was ideated in stage 1. The web-based platform consists of three core components: (1) a dashboard with direct access to appointment scheduling, videoconference sessions, and links to module content; (2) a recording library for practice conversations; and (3) self-directed module content. These core components and the participant journey are depicted in Figure 2.

**Table 3:** Core messages for teaching and learning social communication skills in convers-ABI-lity.

Module	Core message	Submessages
1	We all have some problems with conversations.	A good conversation has many ingredients. A brain injury can change the ingredients that are part of the conversation. Evaluation task: identify strengths and difficulties in one’s own conversations.
2	We can improve our conversations.	We can improve by setting goals. We can improve by working together. We can improve by practicing. We can improve by watching and listening.
3	Match your conversations to the situation.	Match your conversations to where you are talking. Match your conversations to who you are talking to. Match your conversations to who you want to be.
4	Work together to send the message across.	Send the message clearly. Ensure you get the message. Take turns.
5	Talk like you are teammates.	We are in this together (teamwork, equality). We need to support each other (emotional support). I am interested in what you have to say (positive questions and turn-taking).
6	Keep your conversations going.	Start with a good topic. Add in different ways. Add some supports. Find different topics.
7	Make your conversations organized.	Introduce the topic. Organize the parts. Make connections. Wrap it up.

**Table 4:** Differences and similarities in key therapeutic content and processes across TBI Express, TBIconneCT, and convers-ABI-lity: an example using the module “Work Together to Get the Message Across.”

Process or content		TBI Express (synchronous individual and group sessions)	TBIconneCT (synchronous individual sessions)	convers-ABI-lity	
				Synchronous individual sessions	Asynchronous self-guided modules, clinician feedback
**Process**					
	Reflect on personal positive and negative conversation experiences	✓	✓	✓	No asynchronous component
	Discuss completion of home practice	✓	✓	✓	Clinician can review completion of modules before session
	Replay recorded conversation	✓	✓	✓	Clients can access and playback own recordings at any time
	Discuss aspects of conversation	✓	✓	✓	Clinician can annotate practice conversation with timestamped feedback
	Set home practice tasks	✓	✓	✓	Self-guided modules provide structured home practice
	Provide session summary	✓	✓	✓	Session summary stored in platform
	Observe and practice strategies in group format	✓	N/A^a^	N/A	No group format
**Content**					
	Evaluate progress regarding communication goals	✓	✓	✓	No asynchronous component
	Describe communication as a cooperative process between speaker and listener	✓	✓	✓	Self-guided module: web-based video rating tasks
	Discuss speaking strategies	✓	✓	✓	Self-guided module activity: choose personal strategies
	Discuss listening strategies	✓	✓	✓	Self-guided module activity: choose personal strategies
	Discuss turn-taking	✓	✓	✓	Self-guided module activity: rate and then observe how much each person talks in a practice conversation

^a^N/A: not applicable.

### Stage 2: Design Iteratively and Rapidly With User Feedback

#### Overview of Iterative Design

**Figure 3:**
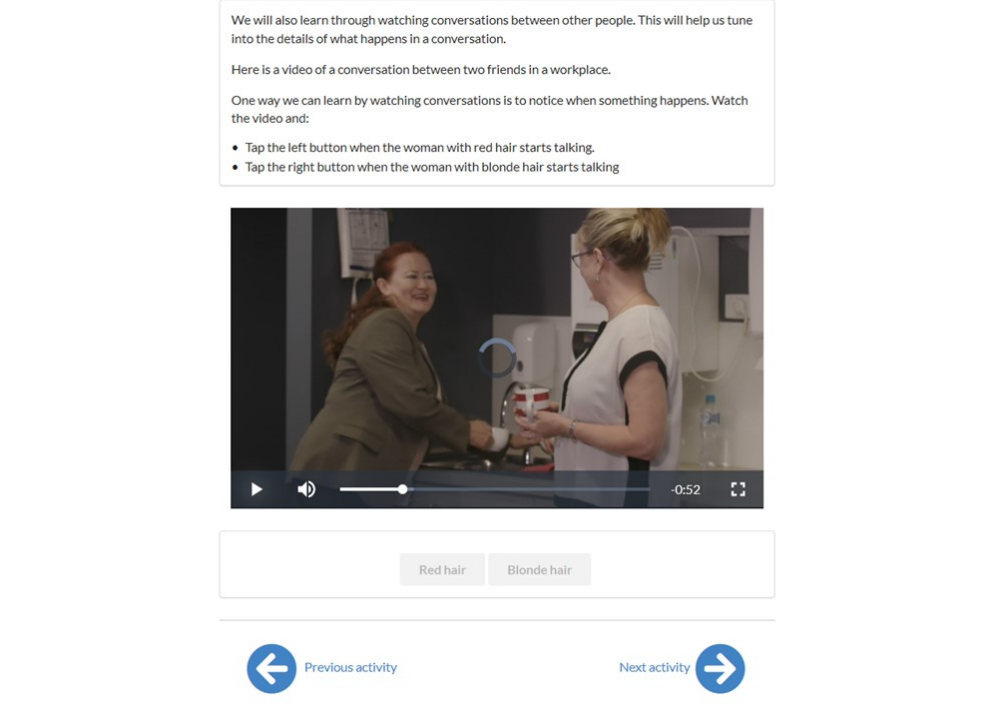
Example of an interactive video task.

Stage 2 addressed the first overall project aim by (1) generating and testing intervention modules, tasks, and features; (2) collecting and analyzing feedback from end users on program features and content; and (3) finalizing the minimum viable product for feasibility testing in stage 3. Following stage 1 (integrate insights from users and theory), content such as web-based video examples and explanations, presentations, quizzes, interactive activities, and reflective questions were developed and incorporated into each module. An example of an interactive video task is depicted in Figure 3. The conversation upload function was built into the platform to allow participants to asynchronously share practice conversations with their speech pathologist and receive feedback. In addition, other features such as appointment bookings and videoconferencing sessions were integrated into the platform, minimizing the need for external tools for both the clinician and clients.

#### User Feedback

##### Participants

A total of 22 participants were interviewed, who had previously participated in interviews focusing on their lived experience and perspectives on the management of cognitive-communication disorders. The results from the initial interviews were used to inform stage 1. The same participants provided feedback on the prototype of convers-ABI-lity. This included people with TBI (n=5); family members of people with ABI (n=4) that included a daughter, a partner, a sister, and a daughter-in-law; speech pathologists (n=4); other health professionals (n=4; including a psychologist, physiotherapist, occupational therapist, and client services manager); and paid support staff (n=5). All 5 participants with ABI were male individuals with severe chronic TBI (median time after injury 6 [range 3-15] years) with the median self-reported posttraumatic amnesia being 77.5 days. Ages ranged from 32 to 63 (median 53) years. Three participants reported vision impairment (not related to the ABI and corrected with glasses), and 1 participant reported a hearing impairment associated with his injury.

Accommodations for the interviews were not necessary. Participants with ABI presented with mild to moderate cognitive-communication disorder as indicated by the FAVRES [56] Task 4 scores. Participants had low-moderate disability as indicated by the median GOS-E score of 5 (range 5-6). The median CANS score was 2 (range 2-4.1), indicating that the participants required support for occupational activities, interpersonal relationships, living skills, or emotional support at least once a week. The family members were all female with a median age of 41 (range 29-54) years. All participants had known a person with ABI for more than 2 years; 2 participants interacted with a person with ABI 3 to 4 times a week, 1 participant interacted with a person with ABI every day, and 1 participant interacted with a person with ABI 1 to 2 days a week. The median years of experience for speech pathologists working in ABI rehabilitation was 15.5 (range 6-20) years and that for other health professionals was 19 (range 10-50) years. The median years of experience for paid support staff was 3 (range 1-25) years. One participant had 25 years of experience in their role; however, they had limited experience working with ABI specifically and reported interacting with a person with ABI less than once a week. Three other paid support staff reported interacting with a person with ABI multiple times per day, and 1 participant reported interacting with a person with ABI a few times a week.

##### Content Analysis

A total of 3 overall categories were derived from the participant interviews: (1) aspects endorsed, (2) suggestions for improvement, and (3) potential applications of convers-ABI-lity (Textbox 2).

Key changes made to the program following participant feedback included: (1) reviewing and editing module names and key messaging to ensure clarity, (2) updating wording from “interview” to “session” throughout the platform, (3) ensuring the appointment date was clear and visible on the client dashboard, (4) ensuring that the module names were written in full on the dashboard, (5) including progress tracking throughout each module (eg, “Step 1 of 6”), (6) simplifying and reducing text to ensure that the information and instructions were client friendly throughout the program, and (7) adjusting features such as automated calculation of speaking time was only visible to the clinician.

**Categories and codes derived from participant interviews on convers-ABI-lity prototype.**

**Endorsed features and content**

- Course features: interactive tasks, integrated recording of practice conversations, automated speaking time calculation, integrated appointment bookings, integrated videoconferencing, simplicity of layout, and access to previous recorded sessions and conversations
- Sense of independence and client control
- Accessibility to specialized services
- Relevance to client and family experience
- Novelty of the program

**Suggestions for improvement**

- Improving wording of some key messages
- Ensuring client friendly language throughout program
- Consideration of culturally and linguistically diverse resources
- Inclusion of a progress bar during completion of a self-guided module
- Consideration of an in-person version to accommodate clients unfamiliar with technology

**Potential applications of convers-ABI-lity**

- Potential for use with other populations (eg, dementia or Parkinson disease)
- Modify platform for other health professions (eg, psychology)
- Potential for international use
- Suitable for telehealth services during and after the COVID-19 pandemic

### Stage 3: Proof-of-Concept Evaluation

#### Participants

A total of 3 participants with ABI and their CPs were recruited to the proof-of-concept study. Participant demographic information and scores on psychosocial, communication, and cognitive assessments are reported in Table 5.

Two of the participants sustained a TBI and 1 participant had a history of bilateral frontal brain tumor (germinoma), with time following injury ranging from 5 to 9 years. Two participants reported vision impairment; P2 presented with blurred vision in the left eye and P3 had no vision in the left eye, poor distance vision, and poor peripheral vision. No hearing impairments were reported. Years of education of the participants ranged from 11 to 15 years. The GOS-E scores indicated lower severe disability for all 3 participants. CANS scores ranged from 6 to 7, indicating participants required support throughout the day for occupational activities, interpersonal relationships, living skills, or emotional support. Moderate to severe cognitive deficits were indicated for all participants based on the RBANS scores, aligning with the FAVRES results that suggested severe cognitive-communication deficits. P3 was unable to complete the FAVRES task due to vision impairment, and additional support was provided to P2 (reading the material out aloud) to assist in task completion. All participants with TBI used the internet every day. Participants did not report receiving other treatment for the duration of the study. All 3 CPs were female and consisted of 2 mothers and 1 paid support worker. The paid support worker had known P1 for less than 2 years and was in contact with P1 for 1 to 2 days a week. P2 and P3 had contact with their CPs every day. CPs of P1 and P2 used the internet every day, whereas P3’s CP used the internet 1 to 2 days a week.

**Table 5:** Proof-of-concept study participant demographic variables.

Demographic variable			Participant 1		Participant 2		Participant 3
Age (years)			22		31		31
Sex			Female		Male		Male
Years of education			11		15		14
Duration after injury (years)			5		5		9
Posttraumatic amnesia			N/A^a^		4 months		Unknown
Type of ABI^b^			Brain tumor		TBI^c^		TBI
Vision impairment			No		Yes		Yes
Hearing impairment			No		No		No
GOS-E^d^ score			Lower SD^e^		Lower SD		Lower SD
CANS^f^ score			7		6		7
**RBANS^g^ score**							
	Immediate memory	40		40		69	
	Visuoconstruction	62		69		84	
	Language	75		74		74	
	Attention	53		43		68	
	Delayed memory	44		40		74	
	Total	49		48		67	
**FAVRES^h^ score**							
	Accuracy SS^i^	1		1		NC^j^	
	Rationale SS	16		16		NC	
	Time SS	102		120		NC	
	Reasoning (raw score)	7		11		NC	
CP^k^ sex			Female		Female		Female
CP age (years)			21		67		65
CP relationship to the person with TBI			Support worker		Mother		Mother
Persons with TBI known to the CP (years)			<2		31		31
Frequency of contact			1-2 d/wk		Every day		Every day
Frequency of internet use of persons with TBI			Every day		Every day		Every day
Frequency of internet use of the CP			Every day		Every day		1-2 d/wk

^a^N/A: not applicable.

^b^ABI: acquired brain injury.

^c^TBI: traumatic brain injury.

^d^GOS-E: Glasgow Outcome Scale–Extended.

^e^Lower SD: lower severe disability.

^f^CANS: Care and Needs Scale.

^g^RBANS: Repeatable Battery for the Assessment of Neuropsychological Status.

^h^FAVRES: Functional Assessment of Verbal Reasoning and Executive Strategies.

^i^SS: standard score.

^j^NC: not completed.

^k^CP: communication partner.

#### Participant Outcomes

##### Primary Outcome Measure: Changes in Conversations Using Objective Rating Scales

The Adapted Kagan Scales were used to objectively measure changes in casual and purposeful conversations. Individual scores are presented in Tables 6 and 7.

For casual conversations, there were no clinically meaningful changes (a change of 0.5 in score) in the MPC Interaction score postintervention for all the 3 participants with TBI. Postintervention, P1 and P2 maintained their preintervention MPC Interaction score and P3 showed regression in their score. However, at follow-up, all the 3 participants showed clinically meaningful positive score changes (an improvement of 0.5) compared with their postintervention scores. For the MPC Transaction score, only P2 demonstrated clinically meaningful change after the intervention. At follow-up, both P1 and P3 showed clinically meaningful score changes compared with postintervention scores; however, the score of P2 decreased by 0.5. For the MSC Acknowledging Competence score postintervention, CP1 and CP2 demonstrated clinically meaningful change compared with their preintervention score. CP3 did not show improvements postintervention for the MSC Acknowledging Competence score. At follow-up for MSC Acknowledging Competence, CP1 and CP3 showed clinically meaningful changes from postintervention scores but not CP2. For the MSC Revealing Competence score, only CP1 had clinically meaningful changes postintervention. All the 3 CPs showed clinically meaningful score changes at follow-up compared with postintervention.

In purposeful conversations, P2 was the only participant with TBI to show clinically meaningful change postintervention for MPC Interaction score. Both P2 and P3 showed an improvement at follow-up; however, P1 did not improve on this measure. For the MPC Transaction score, P1 and P2 improved postintervention compared with preintervention and at follow-up and P2 and P3 showed clinically meaningful change. P1 did not improve at follow-up for this measure. For the MSC Acknowledging Competence score, only CP2 showed improvement postintervention; however, all the 3 CPs showed clinically meaningful changes at follow-up. Similarly, only CP2 showed improvements for the MSC Revealing score postintervention. All the 3 CPs improved on this measure at follow-up. Therefore, at follow-up, 2 of 3 participants with TBI showed increase in interaction and transaction and all the 3 CPs demonstrated increase in either acknowledging or revealing competence.

**Table 6:** Adapted Kagan Scale ratings of casual conversation.

Dyad and time point		Measure of Participation in Conversation			Measure of Support in Conversation	
		Interaction (score out of 4.0)	Transaction (score out of 4.0)	Acknowledging competence (score out of 4.0)		Revealing competence (score out of 4.0)
**1**						
	Preintervention	1.5	1.5	3		3
	Postintervention	1.5	1.5	3.5^a^		3.5^a^
	Follow-up	_3_^a^	_3_^a^	_4_^a^		_4_^a^
**2**						
	Preintervention	1.5	1.5	1.5		1.33
	Postintervention	1.5	_2_^a^	_2_^a^		1.17
	Follow-up	_2_^a^	1.5	2		1.67^a^
**3**						
	Preintervention	2	2.5	2		1.67
	Postintervention	1.5	2	1.5		1
	Follow-up	2.5^a^	2.5^a^	2.5^a^		2.8^a^

^a^Clinically meaningful positive change.

**Table 7:** Adapted Kagan Scale ratings of purposeful conversation.

Dyad and time point		Measure of Participation in Conversation		Measure of Support in Conversation	
		Interaction (score out of 4.0)	Transaction (score out of 4.0)	Acknowledging competence (score out of 4.0)	Revealing competence average (score out of 4.0)
**1**					
	Preintervention	1	1	2.5	3.17
	Postintervention	1	1.5^a^	2.5	2.67
	Follow-up	0.5	1	3.5^a^	3.33^a^
**2**					
	Preintervention	0.5	0.5	1.5	1.5
	Postintervention	1.5^a^	1.5^a^	_2_^a^	1.83^a^
	Follow-up	2.5^a^	2.5^a^	_3_^a^	_3_^a^
**3**					
	Preintervention	1.5	1.5	1.5	1.83
	Postintervention	1	1.5	1	1
	Follow-up	2.5^a^	_2_^a^	_2_^a^	2.17^a^

^a^Clinically meaningful positive change.

##### Secondary Outcome Measure: Self-Report and Significant Other–Report Measures

Table 8 shows the data for the secondary communication outcome measure, the LCQ, as reported by the person with TBI and their CP. There were no statistically reliable changes on the LCQ total for both the self-report and significant other–report between preintervention and postintervention or postintervention and follow-up.

Table 9 shows the data for the quality-of-life measures for the person with TBI and their CP. P1 reported a statistically reliable positive change on the QOLIBRI cognition scale between preintervention and postintervention. At postintervention, P2 reported a statistically reliable negative change in his daily life and autonomy (ie, toward less autonomy). P3 did not show any statistically reliable changes across all QOLIBRI scales postintervention or at follow-up. P1 did not show any statistically reliable differences postintervention or at follow-up on the TBI-CareQOL scores. CP2 reported statistically reliable negative change on the scale of anxiety (ie, increase in anxiety) and a statistically reliable positive change on the scale of loss of self (ie, improved sense of self). There were no further statistically reliable differences for this participant. CP3 did not report any significantly reliable differences postintervention for health-related quality of life

Participant raw scores for the SPRS-2 are reported in Table 10. At postintervention, P1 reported improvements in the domains of work and leisure and living skills. They did not complete the questionnaire for the domain of relationships preintervention; therefore, a total score was not calculated for this time point for comparison with postintervention scores. At 3-month follow-up, P1 reported improvements in the domains of work and leisure and relationships. They reported negative changes for living skills domain; however, there was an overall improvement at follow-up on the total score. P2 did not report any change in the domains of work and leisure and living skills postintervention compared with that of preintervention. It was unclear why P2 did not complete the relationships domain at preintervention; as a result, the scores for this domain and the total score were not able to be compared with the postintervention scores. At follow-up, P2 reported further improvements in the domain of work and leisure and no change in the domain of relationships compared with those postintervention. The living skills domain was reported to be worse at follow-up compared with that preintervention; however, there was overall improvement in the total score. At postintervention, P3 reported no change in the domain of work and leisure and negative changes in the domains of relationships and living skills compared with those preintervention. There was a negative change in the total score between preintervention and postintervention. At follow-up, P3 reported negative changes for the domains of work and leisure and relationships; however, there was an improvement in the living skills domain compared with that postintervention. There was a negative change of 1 for the total score at follow-up assessment.

**Table 8:** La Trobe Communication Questionnaire (LCQ) scores.

Participant		Preintervention assessment^a^	Postintervention assessment^a^	Follow-up^a^	Pre- to postintervention change^b^ (RCI^c^),	Postintervention to follow-up change^d^ (RCI)
**LCQ (self): total score^e^** **(out of 120)**						
	P1	37	33	38	−4 (−0.46)	+5 (0.58)
	P2	66	51	42	−15 (−1.74)	−9 (−1.04)
	P3	47	46	53	−1 (−0.12)	+7 (0.81)
**LCQ** **(other): total score** ^e^ **(out of 120)**						
	P1	56	54	47	−2 (−0.26)	−7 (−0.92)
	P2	51	53	50	+2 (0.26)	−3 (−0.39)
	P3	63	57	55	−14 (−0.78)	−2 (−0.26)

^a^Raw scores.

^b^Difference in scores between pre- and postintervention.

^c^RCI: reliable change index.

^d^Difference in scores between post and follow-up.

^e^Note that lower scores represent less communication difficulty.

**Table 9:** Quality of Life after Brain Injury (QOLIBRI) and Traumatic Brain Injury Caregiver Quality of Life (TBI-CareQOL) scores and reliable change index (RCI).

Participant and CP^a^			Preintervention assessment^b^		Postintervention assessment^b^		Follow-up assessment^b^		Pre- to postintervention change^c^ (RCI)		Postintervention to follow-up change^d^ (RCI)
**QOLIBRI-cognition**											
	P1	46.5		96.43^e^		82.14		+49.93 (3.72)^f^		−14.29 (−1.06)	
	P2	67.86		64.25		67.86		−3.61 (−0.27)		+3.61 (0.27)	
	P3	78.5		71.5		82.14		−7 (−0.52)		+10.64 (0.79)	
**QOLIBRI-self**											
	P1	42.75		NC^g^		89.28		N/A^h^		N/A	
	P2	89.29		67.75		53.57		−21.54 (−1.73)		−14.18 (−1.14)	
	P3	46.5		67.75		67.86		+21.25 (1.71)		+0.11 (0.01)	
**QOLIBRI–d** **aily life and autonomy**											
	P1	92.75		89.29		96.43		−3.46 (−0.27)		+7.14 (0.55)	
	P2	67.86		17.75		28.57		−50.11 (−3.84)^i^		+10.82 (0.83)	
	P3	21.5		17.75		28.57		−3.75 (−0.28)		+10.82 (0.83)	
**QOLIBRI–social relationships**											
	P1	95		100		100		+5 (0.34)		0 (0)	
	P2	58.33		66.5		41.67		+8.17 (0.57)		−24.83 (−1.69)	
	P3	33.25		50		50		+16.75 (1.14)		0 (0)	
**QOLIBRI–emotions**											
	P1	100		100		100		0 (0)		0 (0)	
	P2	75		90		100		+25 (0.92)		+10 (0.61)	
	P3	70		70		60		0 (0)		−10 (−0.61)	
**QOLIBRI–physical problems**											
	P1	100		100		100		0 (0)		0 (0)	
	P2	65		45		50		−20 (−1.51)		+5 (0.38)	
	P3	65		50		50		−15 (−1.13)		0 (0)	
**TBI-CareQOL–anxiety**											
	CP1	33.92		33.92		33.92		0 (0)		0 (0)	
	CP2	33.92		53.46		48.07		+19.54 (4.01)^i^		−5.39 (−1.11)	
	CP3	43.35		46.62		41.28		+3.27 (0.67)		−5.34 (−1.1)	
**TBI-CareQOL–strain**											
	CP1	32.13		32.13		32.13		0 (0)		0 (0)	
	CP2	43.14		36.93		39.21		−6.21 (−0.19)		+2.28 (0.44)	
	CP3	46.31		47.78		44.78		+1.47 (0.28)		−3 (−0.58)	
**TBI-CareQOL–trapped**											
	CP1	37		37		37		0 (0)		0 (0)	
	CP2	50.41		57.74		53.6		+7.33 (1.50)		−4.14 (−0.85)	
	CP3	52.55		53.6		51.49		+1.05 (0.20)		−2.11 (−0.43)	
**TBI-CareQOL–loss of person with traumatic brain injury**											
	CP1	31.14		35.87		35.24		+1 (0.22)		−0.63 (−0.14)	
	CP2	54.03		56.32		40.62		+2.29 (0.50)		−15.7 (−3.38)^i^	
	CP3	51.75		47.05		44.54		−4.7 (−1.02)		−2.51 (−0.54)	
**TBI-CareQOL–loss of self**											
	CP1	35.24		35.24		31.14		0 (0)		−4.1 (−0.14)	
	CP2	52.97		40.62		50.61		−12.35 (−2.66)^i^		+9.99 (2.18)^i^	
	CP3	46.47		40.62		44.87		−15.85 (−1.26)		+4.25 (0.93)	

^a^CP: communication partner.

^b^Raw scores.

^c^Difference in scores between pre- and postintervention.

^d^Difference in scores between post and follow-up.

^e^Note that higher scores represent improved outcomes on all measures except TBI-CareQOL.

^f^Statistically reliable positive difference.

^g^NC: not completed.

^h^N/A: not applicable.

^i^Statistically reliable negative difference.

**Table 10:** Sydney Psychosocial Reintegration Scale 2 (SPRS-2) raw scores.^a^

SPRS-2 domains and participant			Preintervention assessment^b^		Postintervention assessment^b^		Follow-up assessment^b^		Pre- to postintervention change^c^		Postintervention to follow-up change^d^
**Work and leisure**											
	P1	9		12		13		+3		+1	
	P2	5		5		7		0		+2	
	P3	2		2		1		0		−1	
**Relationships**											
	P1	NC^e^		11		13		N/A^f^		+2	
	P2	NC		12		12		N/A		0	
	P3	8		7		6		−1		−1	
**Living skills**											
	P1	11		13		12		+2		−1	
	P2	7		11		10		+4		−1	
	P3	8		4		5		−4		+1	
**Total**											
	P1	N/A		36		38		N/A		+2	
	P2	N/A		28		29		N/A		+1	
	P3	18		13		12		−5		−1	

^a^Higher scores represent improved participation.

^b^Raw scores.

^c^Difference in scores between pre- and postintervention.

^d^Difference in scores between post and follow-up.

^e^NC: not completed.

^f^N/A: not applicable.

#### Feasibility

All participants completed an initial assessment session and all 7 sessions of convers-ABI-lity, within 7 weeks. The average self-guided module completion percentage ranged between 81% and 100% completion. P1 completed 100% of the module tasks, P2 completed 92% of the module tasks, and P3 completed 81% of the module tasks.

#### Participant Feedback

Dyads completed the postintervention interviews together, with interview duration ranging between 21:26 and 37:22 minutes. Through content analysis, three categories were derived: (1) aspects endorsed, (2) challenges of the course, and (3) recommendations for improvement. These are presented in Table 11.

Participants endorsed the engaging program content and features and perceived as they were relevant to their lived experience. Some participants reported about the challenges with the program including technical difficulties due to unfamiliarity with technology and feelings of discomfort when recording practice conversations but still identified their positive use. One participant (CP1) suggested that some tasks may be too challenging for some people with TBI because of the multitasking nature of the activity. Recommendations for improvement included increasing the number of sessions, completing the program with paid support workers, and resolving technical issues within the platform. Participant feedback from the earlier development stage and this proof-of-concept study guided changes that will be implemented in the next iteration of convers-ABI-lity, with the aim to evaluate this revised version in a larger pilot study.

**Table 11:** Categories, codes, and participant quotes from feedback interviews postintervention

Category, subcategory, and code				Exemplar quotes from participants and communication partners
**Aspects endorsed**				
	**Intervention features**			
		Novelty of the program (eg, engaging) Connection to the platform and navigation of the platform is easy Appropriate length and frequency of sessions Opportunities to practice conversations and review recorded practice conversations	“I think time flew by... because it was very interesting and engaging” [CP1^a^] “Answering questions, watching videos was perfect” [P2] “We would watch back our videos, and talk about what we did well and what we do to make conversations better... having that visual was really good to then implement those strategies to make conversations better.” [CP1]	
	**Intervention content**			
		Self-guided modules were relevant to client experience Relevant and useful content covered throughout the program Learning outcomes	“This study changed me... dramatically, enormously. And because of me, I can now do much more for my son” [CP2] “When I had the answers [from P2], I didn’t ask more. It was enough. But now I can understand it’s not enough. I try to ask why, when how do you think about this.” [CP2] “Passing the ball... Talk as a team member, not a coach.” [P3]	
	**Intervention delivery**			
		Synchronous sessions consolidated self-directed content Collaboration between dyad and clinician Clear explanations and delivery of content	“I think [the videoconference sessions] really put the module of each week into perspective and practice, and it made the modules a lot more understandable.” [CP1] “I like the way how [clinician] delivered everything. Found our problems...found the strategies.” [CP2]	
**Challenges of the course**				
	**Experience with technology**			
		Technical challenges for those unfamiliar with technology	“I just found it on the technical side frustrating at times, and I’d often call on my daughter who is more tech savvy to get the program up and running... Once you started the session it was fine.” [CP3]	
	**Personal preferences and challenges**			
		Feeling uncomfortable recording conversations on camera Some self-directed tasks required multitasking, such as interactive videos (challenging for person with TBI^b^)	“Recording conversations was no good.” [P2] “that was probably a little bit more difficult to do that [interact with video]... multitask while watching the video [for person with brain injury]” [CP1]	
**Recommendations for improvement**				
	**Recommendations**			
		Increasing number of sessions May be better completed with support workers rather than family Resolving technical issues in the self-guided modules	“I think it’s not enough [sessions]... we just started to understand” [CP2] “[have sessions] Every day, because it would be fresh in your mind” [P2] “it’s not my sort of a program, I was hoping he could do it with his support worker” [CP3] “once you actually got into the homework... there were a few little glitches” [CP3]	

^a^CP: communication partner.

^b^TBI: traumatic brain injury.

## Discussion

### Principal Findings

The overall objective of this study was to outline the development process and evaluate a novel web-based CPT intervention called convers-ABI-lity for people with ABI and their CPs. An iterative and collaborative approach with key stakeholders was used throughout the design and development stages. This approach resulted in a digital health intervention that people with ABI and their CPs qualitatively reported to be relevant to the client experience, contain engaging and useful content and features, and facilitate a sense of independence and client control over their therapy. Quantitative evaluation of participant outcomes also indicated promising findings for using convers-ABI-lity to improve conversations after ABI. The first research question sought to determine whether convers-ABI-lity produced changes in conversations between the person with ABI and their CP. The results from the proof-of-concept study demonstrated that all participants made some improvements in conversation skills as measured by the Adapted Kagan Scales [61], consistent with findings from existing programs, TBI Express [17], and TBIconneCT [24,25]. All 3 participants with ABI showed improvements in their participation in conversation at follow-up compared with that before the intervention for casual conversations, and 2 participants showed improvements at follow-up compared with that before the intervention for purposeful conversations. Similarly, CPs were more likely to demonstrate clinically meaningful score changes in their ability to provide support during a conversation at follow-up than after the intervention when compared with the preintervention scores. The finding that meaningful changes were evident in follow-up rather than immediately postintervention may suggest that increased time and opportunities to practice and consolidate new learnings and strategies are required for both people with ABI and their CPs to improve their conversation skills. Opportunities for increased practice were endorsed by participant feedback after the intervention. In addition, all participants had clinically meaningful score change at follow-up for casual and purposeful conversations, indicating that the convers-ABI-lity program has the potential to improve conversations after ABI. The second research question investigated if participants improved on self-report and significant other–report measures (such as family member report) related to changes in their communication. All participants except for CP2 reported some improvements for the person with ABI in social communication skills in conversation between before the intervention and after the intervention but not at a magnitude to represent a statistically reliable change in these individuals. At both time points, before and after the intervention, participants with ABI were more likely to report fewer occurrences of social communication deficits than their CPs. At follow-up, all CPs reported improvements in social skills for the person with ABI. P1 and P3 reported higher frequencies of communication deficits at follow-up than at postintervention. However, the scores at follow-up were closer to those of their respective CPs. Reduced insight and awareness are associated with brain injury [74], often impacting the recognition of communication breakdown and appropriate repair of conversation for people with cognitive-communication disorders [75]. These results suggest that participants with ABI made potential improvements in insight into their cognitive-communication difficulties during the postintervention phase.

The third research question evaluated if there were statistically reliable changes on self-reported quality of life measures for the participants with ABI and their CP. Although there were few statistically reliable changes across the measures, participant raw scores did indicate small improvements across different time points. For participants with ABI, there were individual improvements in health-related quality of life domains of the sense of self and social relationships. Other personal contextual factors such as COVID-19 pandemic lockdowns, physical and mental health, and scheduled surgeries may have impacted the outcomes of these measures, which were not directly linked to communication function. CPs also had some small improvements in the raw scores, reporting fewer feelings of loss of self after the intervention and feeling less trapped at follow-up. The role of being the primary CP can carry substantial caregiver burden, particularly during challenging times in a global health crisis. Evaluating quality of life changes in relation to changes in cognitive-communication function is an essential process to understanding the impact of communication on the lives of people with ABI and their CPs. However, it is crucial to consider the person within a more holistic context and the complex interplay among personal, familial, community, and global factors. The use of these measures was demonstrated to be useful for data collection and therefore appropriate for the evaluation of the efficacy of convers-ABI-lity in larger studies.

Finally, this study evaluated the feasibility of delivering convers-ABI-lity via web-based platforms and investigated how the program was perceived by the participants who completed the intervention. All participants completed all 7 sessions of the intervention program. Self-guided weekly modules were also completed to a high degree suggesting that participants were engaged and motivated with the content and weekly tasks. Only 1 CP reported challenges and frustrations with using the technology and felt that the web-based intervention program was not suitable for her. Despite these sentiments, the CP and their son with TBI did achieve positive outcomes for communication skills after the intervention and at follow-up, as measured by objective assessment and self-report. Other participants reported no barriers connecting to or navigating the platform. This finding is consistent with participant feedback given by key stakeholders on the convers-ABI-lity prototype where they identified that digital interventions may not be suitable for everyone and in-person intervention tools still have a place in the clinical toolkit. Therefore, clinicians need to consider client familiarity and comfort with using technology during their clinical decision-making in choosing the most appropriate intervention tool.

Participants (across both stage 2 and stage 3 studies) valued the complementary nature of the asynchronous self-directed modules and the synchronous sessions with the clinicians. They reported that the clinician sessions allowed for opportunities to consolidate the new knowledge from the modules and practice the implementation of communication strategies. However, participants perceived the program would benefit from additional clinician sessions to further synthesize and consolidate learning outcomes and communication skills, often feeling that they were just beginning to understand and grasp new concepts as the program was finishing. Many features of the program such as interactive videos, automated speaking time calculations, access to recorded practice conversations and previous sessions, integrated recording of practice conversations, appointment bookings, and videoconferencing were endorsed by the participants in stages 2 and 3 of the project. However, 1 CP who completed the intervention in stage 3 indicated that some interactive tasks, such as watching a video and clicking a button when a specified communication behavior was observed, necessitated multitasking skills that were too challenging for a person with TBI. This difficulty may be attributed to impaired cognitive function experienced by people with TBI and may further emphasize the need for this program to be completed with the support of a CP. Finally, 2 participants reported feeling uncomfortable recording their conversations on camera with 1 of the participants also feeling particularly uncomfortable with reviewing the practice conversations. Discomfort could potentially influence the participants’ performance during the conversation tasks, for example, reducing their level of participation or support in conversation. Regardless, both participants still reported that they found the program useful and would recommend it to others. The selection of intervention tools needs to be complementary to the needs of the clients. Interventions with digital health features can be engaging, flexible, and more accessible to people with TBI and their CPs. However, it is important for clinicians to consider the preferences of their clients and choose tools that are most appropriate for facilitating positive outcomes.

### Comparisons With Prior Work

This is the first study on CPT following ABI to apply an a priori approach to collaborative intervention design and development. Existing programs such as TBI Express [17] and TBIconneCT [24,25] were integral in informing the first stage of this study to form the foundations of convers-ABI-lity. Qualitative feedback collected formally following intervention on participant perceptions [21,26] and informally from clinicians implementing these programs into clinician services assisted in conceptualizing the next steps for digital health adaptation. The development of these existing interventions followed a more traditional approach, whereby intervention development initially relied on expert opinion, and participant viewpoints were sought following the creation of the intervention [74,76]. For the development of convers-ABI-lity, the authors applied an iterative co-design approach from the conception of the project. This allows for the continual refinement of the intervention program with iterative end-user feedback and input, accelerating the development of an effective digital health intervention. In the landscape of fast-evolving technology, this approach is preferred to traditional approaches as intervention development can keep pace with technological advancements through the dynamic nature of design and development.

Through the collaborative design process, some key changes were integrated into convers-ABI-lity from the original CPT programs. One key change is the reduced direct clinician time required. TBI Express comprises 35 clinician-directed hours, which was reduced to 15 hours in TBIconneCT. Direct clinician time in convers-ABI-lity amounts to approximately 8 hours. The sessions have also been reduced in length from 1.5 hours to 1 hour. Despite the reduction in direct clinician hours, it is anticipated that treatment dose remains relatively high as clients complete 1 to 2 hours of asynchronous tasks during each intervention week. The process measures in the proof-of-concept study indicate that the participants were willing to engage with this self-directed content and completed it to a high degree, suggesting that treatment dosage can be maintained without direct clinician time.

Although there has been reduction in direct clinician hours, the inclusion of asynchronous client tasks and prerecorded practice conversations does necessitate indirect clinician time when reviewing client progress during the week. It is recommended that future research investigates treatment dose as delivered by convers-ABI-lity and indirect clinician time, which can have implications for service delivery and client funding and fees.

The completion of self-directed content may be challenging for individuals with cognitive and cognitive-communication impairments. Participant feedback following the proof-of-concept study indicated that people with brain injury may require additional support from the CP to understand and complete self-guided tasks. This recommendation aligns with a contextualized rehabilitation approach [77,78], whereby CPs and people with cognitive-communication disorders work collaboratively to complete tasks and improve conversation skills. This philosophy is grounded in the theoretical underpinnings that have been carried over to convers-ABI-lity from TBI Express and TBIconneCT. These approaches of collaboration and cognitive support are highly recommended intervention components of cognitive-communication rehabilitation [12,79]. The use of the IDEAS framework [43] ensured that evidence-based treatment ingredients and underlying theoretical foundations were transferred into the web-based environment to promote intervention effectiveness.

### Study Limitations

Given that the intended target audience for convers-ABI-lity is people with ABI, participants with nonprogressive brain injuries such as brain tumors, hypoxic brain injury, stroke, poisoning, and infection were eligible for this study. However, the participants included across all study phases were people with TBI (with the exception of 1 participant in stage 3 study who experienced a nonprogressive brain tumor). The lack of representation of people with other types of ABI may affect the generalizability of the findings from this study to the wider population with ABI that may experience cognitive-communication disorders. Furthermore, the participants in this research were predominantly male individuals with TBI, further limiting the generalizability of the findings to female individuals with TBI. It is important to acknowledge the small sample size in this proof-of-concept study, warranting further research with a larger sample of people with brain injury and CPs to determine treatment efficacy and effectiveness. Participants self-selected to be included in the research, potentially demonstrating higher levels of motivation to engagement than may be representative in the clinical population. Potential participant fatigue, reduced attention, and time limitations may have affected the provision of feedback on convers-ABI-lity during the interview phases.

Participant demographic data such as cultural background or languages spoken were not formally collected and materials and resources presented during interviews and intervention were presented in English only. The consideration of culturally and linguistically diverse processes and materials is an important consideration for future research. Finally, the completion of questionnaires via self-guided modules in the intervention does improve administration time efficiency; however, the responses may be less accurate than those acquired from an interview approach.

### Study Implications and Lessons Learned

This study is innovative in 2 regards. First, it is the first study in cognitive-communication rehabilitation to outline the development of CPT intervention with iterative key stakeholder input throughout the process. The stages of development and evaluation detailed in this study may be of interest to others working in the development of digital health interventions, particularly for behavioral, cognitive, or communication-based interventions. Second, the preliminary findings for convers-ABI-lity treatment outcomes suggest positive results for improving conversation skills of people with TBI and their CPs. Further evaluation of treatment effectiveness and efficacy using these outcome measures is required within a larger study, particularly within a clinical context with rehabilitation clinicians.

Through the development and early evaluation process of convers-ABI-lity, a number of key lessons emerged:

- A collaborative and iterative approach with end users to digital health intervention development promotes relevance and usefulness of the intervention, as a result of incorporating content and activities that more accurately reflect the clients’ lived experiences.
- Content, activities, and features need to be accessible for all end users from a communication perspective such as CPs, clinicians, and people with cognitive-communication disorder. An approach that empowers the person with ABI as an equal participant during the intervention process can be achieved with the use of clear and noncomplex language while presenting intervention information and instructions.
- This study trialed outcome measures to determine suitability of their use in a larger clinical trial of this intervention. This phased approach to the development and evaluation of complex interventions is recommended by the Medical Research Council [77]. These outcome measures addressed each domain of the International Classification of Functioning [33] and have been shown to be suitable for use in future research.
- Digital health interventions may not be a replacement for existing in-person interventions but an additional option for clinicians to consider when selecting intervention tools that best meet client preferences and needs.

### Conclusions

This is the first study in the field of cognitive-communication rehabilitation in ABI to use a collaborative design approach in developing and evaluating a novel multimodal web-based CPT program. The results of this study indicate that it is beneficial to work collaboratively and iteratively with key stakeholders in the design of an intervention program, with preliminary results showing improvements in conversations of people with ABI and their CPs. This innovative web-based multimodal format offers new opportunities for greater access to specialist rehabilitation services for people with ABI and improved flexibility of service delivery for clinicians. Further research with a larger sample size is warranted to further investigate the efficacy and effectiveness of this newly developed web-based intervention program.

## Appendix

Multimedia Appendix 1 Good reporting of a mixed methods study (GRAMMS) checklist.

Multimedia Appendix 2 Interview guide for participant feedback on convers-ABI-lity prototype content and features.

Multimedia Appendix 3 Interview guide for participant feedback following completion of convers-ABI-lity in proof-of-concept study.

Multimedia Appendix 4 Worked example of coding.

Multimedia Appendix 5 Adapted Kagan Scales description.

## Abbreviations

ABI: acquired brain injury
CANS: Care and Needs Scale
CP: communication partner
CPT: communication partner training
FAVRES: Functional Assessment of Verbal Reasoning and Executive Strategies
GOS-E: Glasgow Outcome Scale–Extended
ICC: intraclass correlation coefficient
IDEAS: Integrate, Design, Assess, Share
LCQ: La Trobe Communication Questionnaire
MPC: Measure of Participation in Conversation
MSC: Measure of Support in Conversation
QOLIBRI: Quality of Life after Brain Injury
RBANS: Repeatable Battery for the Assessment of Neuropsychological Status
RCI: reliable change index
SPRS-2: Sydney Psychosocial Reintegration Scale 2
TBI: traumatic brain injury
TBI-CareQOL: Traumatic Brain Injury Caregiver Quality of Life
