# Supplementary material for: Developing a Digital Health Intervention for Conversation Skills After Brain Injury (convers-ABI-lity) Using a Collaborative Approach: Mixed Methods Study
Source: J Med Internet Res. 2023 Aug 9;25:e45240. doi: 10.2196/45240 (PMC10448295; doi:10.2196/45240)
Supplement: Multimedia Appendix 1 [file jmir_v25i1e45240_app1.docx]

**Multimedia Appendix 1**

*Good Reporting of a Mixed Methods Study (GRAMMS) checklist*

Developed from: O’Cathain, A., Murphy, E., & Nicholl, J. (2008). The Quality of Mixed Methods Studies in Health Services Research. *Journal of Health Services Research and Policy, 13*(2), 92–98. https://doi.org/10.1258/jhsrp.2007.007074

| Guideline | Reported on page no. |
| --- | --- |
| Describe the justification for using a mixed methods approach to the  research question | p. 8 |
| Describe the design in terms of the purpose, priority and sequence of  methods | p. 8-10, p. 13-15 |
| Describe each method in terms of sampling, data collection and  analysis | p. 11-13, p. 16-21 |
| Describe where integration has occurred, how it has occurred and  who has participated in it | p. 21-32, p. 39-42 |
| Describe any limitation of one method associated with the present of  the other method | p. 49-50 |
| Describe any insights gained from mixing or integrating methods | p. 43-49, p. 50-52 |
