# Supplementary material for: Developing a Digital Health Intervention for Conversation Skills After Brain Injury (convers-ABI-lity) Using a Collaborative Approach: Mixed Methods Study
Source: J Med Internet Res. 2023 Aug 9;25:e45240. doi: 10.2196/45240 (PMC10448295; doi:10.2196/45240)
Supplement: Multimedia Appendix 2 [file jmir_v25i1e45240_app2.docx]

**Multimedia Appendix 2**

*Interview guide for participant feedback on convers-ABI-lity prototype content and features.*

Convers-ABI-lity is designed as a therapeutic platform for the person with TBI and their communication partner to link in with a Speech Pathologist using online teleconferencing and complete self-directed activities to support and improve their communication after brain injury.

This is a 10-week intervention program. Due to our time limitations, I will show you some screenshots from the program which include some of the different features we have incorporated.

As I show you and talk through each slide, please provide as much feedback as you can.

SLIDE 1 (Modules, booking requests and schedule)

These are some things you may see on the home page of convers-ABI-lity. On your left here, you can see the modules that contain activities which the client and their carer can do on their own.

This bottom section has links for the client to join the scheduled videoconference with the speech pathologist. You can also see when your next session is coming up.

In this middle section, you can request to book in a session with your speech pathologist and the “previous interviews” link allows you to view your recordings and notes from previous sessions.

QUESTIONS:

- What are your first impressions? Feel talk through your thoughts as much as you can
- What’s something you like about what you see here?
- What is something you think we could change?

SLIDE 2: (module titles)

Next, we’ll have a look at the module titles. *Interviewer to read each one out, slowly and clearly.*

QUESTIONS:

- What are your first impressions of the module titles?
- Is there anything that stands out to you?
- Is there something that could make it sound or read better?

SLIDE 3: (Activity – uploading a video)

This is an example of one type of activity where you can record conversations as a practice task. We can have a look at the instructions together.

The conversation recordings are uploaded for the speech pathologist to watch and give you feedback.

Something else to note while we are going through these examples is that at the top of each activity, you can see how many steps there are in a module and the progress that you have made.

There are also back and next buttons at the bottom of the screen.

QUESTIONS:

- What are your first impressions? Feel talk through your thoughts as much as you can
- What’s something you like about what you see here?
- What is something you think we could change?

SLIDE 4 (Video)

We have also incorporated short 1-2 minute videos or animations to teach new ideas. Unfortunately this is just a screenshot and we can’t watch it at the moment.

QUESTIONS:

- What are your first impressions? *Feel talk through your thoughts as much as you can*
- What’s something you like about what you see here?
- What is something you think we could change?

SLIDE 5 (Interactive videos)

There are also interactive videos where you can do a task while you watch. Let’s have a look at the instructions for this video.

At the end, you will be able to see how much each person contributed to the conversation.

QUESTIONS:

- What are your first impressions? *Feel talk through your thoughts as much as you can*
- What’s something you like about what you see here?
- What is something you think we could change?

SLIDE 6 (Personal response activities)

Some activities are about giving a personal response. These answers are uploaded so the speech pathologist can see your responses. It may be something you can then talk about further in your session.

QUESTIONS:

- What are your first thoughts on this?
- What do you like about this activity?
- Is there something you feel we could improve?

SLIDE 7 (SP view)

This is an example of what the speech pathologist sees during the videoconference. They can mark certain moments in the video using this annotate feature and they can also write notes for the client to see after the session.

QUESTIONS:

- Share your thoughts about this screen or these features
- What’s something that you find interesting or useful?
- What’s something that we could do differently?

SLIDE 8 (client view)

This is our final slide. This is what the client sees. They can see a side-by-side view of the videoconference recording.

The amount of talking tie for each person is automatically calculated and recorded in this section.

The client can also view moments marked by the speech pathologist and they can read the speech pathologist’s notes in this bottom section.

QUESTIONS:

- Share your thoughts about this screen or these features
- What’s something that you find interesting or useful?
- What’s something that we could do differently?
