# Supplementary material for: Developing a Digital Health Intervention for Conversation Skills After Brain Injury (convers-ABI-lity) Using a Collaborative Approach: Mixed Methods Study
Source: J Med Internet Res. 2023 Aug 9;25:e45240. doi: 10.2196/45240 (PMC10448295; doi:10.2196/45240)
Supplement: Multimedia Appendix 3 [file jmir_v25i1e45240_app3.docx]

**Multimedia Appendix 3**

*Interview guide for participant feedback following completion of convers-ABI-lity in proof-of-concept study*

Questions for person with ABI and their communication partner (interview conducted jointly). These questions were accompanied by a PowerPoint presentation with key words and visual supports (e.g. pictures or graphics) to aid comprehension.

1. When you started two months ago How did you find the process of getting started with the program?
2. Week by week you had video sessions with Rachael. What did you think about those?
3. You worked with Rachael to figure out some “communication tips”. What did you think about these tips?
4. You had some activities to complete on your own. What did you think about them?
5. What did you think about the different activity types? (e.g., watching videos, answering questions and recording conversations)

*Timing:*

1. What did you think about the number of sessions (8)?
2. What did you think about the length of session (45-60 minutes)
3. What did you think about how often the sessions took place (every week)?

*Goals:*

1. Think back to what you hoped you would learn in this course. How well did the course cover these things?

*This course was about improving conversations:*

1. Have there been any improvements for the two of you?
2. If so - could you share an example of the most significant change in your conversations?

*The future:*

1. How are you planning to keep working on conversations?
2. Did the course give any ways to keep working on conversations on your own?

*Finishing questions:*

1. What would you say to someone who was thinking about doing this program?
2. How useful was this course for you? (present 5-point scale; from extremely useful to not useful at all)
3. How likely would you be to recommend this course to other families? (Present 10-point scale
4. Would you like to share further reflections about being involved in this research?
