# Supplementary material for: Developing a Digital Health Intervention for Conversation Skills After Brain Injury (convers-ABI-lity) Using a Collaborative Approach: Mixed Methods Study
Source: J Med Internet Res. 2023 Aug 9;25:e45240. doi: 10.2196/45240 (PMC10448295; doi:10.2196/45240)
Supplement: Multimedia Appendix 4 [file jmir_v25i1e45240_app4.docx]

**Multimedia Appendix 4**

*Worked example of coding*

| Speaker | Transcript | Meaning unit | Code |
| --- | --- | --- | --- |
|  |  |  |  |
| Interviewer | [Presenting slide 8] and this is what the client sees. They can see a side-by-side view of the videoconference recording. The amount of talking time for each person is automatically calculated and recorded in this section. |  |  |
| P3 | Oh that’s good, because it encourages at the end of the day, when you see it back, whether they’ve had much input. Yep, that’s good. | That’s good, because it encourages you to see if they have had much input | Positive comment re: automatic calculation of talking time |
| Interviewer | The client can also view moments marked by the speech pathologist and they can read the speech pathologist’s notes in this bottom section. So what are your thoughts about these screens? |  |  |
| P3 | It’s a good tool, definitely. I guess the screen’s pretty helpful. Like I said, whilst you’re conducting the interview, it’s good to see it all there in front of you, so yeah. | It’s a good tool… it’s good to see it all there in front of you. | Positive feedback re: client view of recorded VC session |
