# Supplementary material for: Developing a Digital Health Intervention for Conversation Skills After Brain Injury (convers-ABI-lity) Using a Collaborative Approach: Mixed Methods Study
Source: J Med Internet Res. 2023 Aug 9;25:e45240. doi: 10.2196/45240 (PMC10448295; doi:10.2196/45240)
Supplement: Multimedia Appendix 5 [file jmir_v25i1e45240_app5.docx]

**Multimedia Appendix 5**

*Adapted Kagan Scales description*

| Rating scale | Area of assessment | Description |
| --- | --- | --- |
|  |  |  |
| Measure of Participation in Conversation (MPC) | Transaction | The person with TBI takes responsibility for sharing the conversation interaction appropriately |
|  | Interaction | The person with TBI conveys content and shows understanding of the conversation |
| Measure of Support in Conversation (MSC) | Acknowledging competence | The communication partner talks in a natural adult way and demonstrates sensitivity to the person with TBI |
|  | Revealing competence | The communication partner ensures the person with TBI can understand and respond during conversation  The communication partner uses behaviours to ensure they understand the person with TBI |
